# Using dynamic *N*-mixture models to test cavity limitation on northern flying squirrel demographic parameters using experimental nest box supplementation

**DOI:** 10.1002/ece3.1086

**Published:** 2014-05-05

**Authors:** Pauline Priol, Marc J Mazerolle, Louis Imbeau, Pierre Drapeau, Caroline Trudeau, Jessica Ramière

**Affiliations:** 1 Chaire industrielle CRSNG-UQAT-UQAM en Aménagement Forestier Durable, Institut de Recherche sur les Forêts, Université du Québec en Abitibi-Témiscamingue445 boul. de l'Université, Rouyn-Noranda, QC, J9X 5E4, Canada; 2 Centre d'Étude de la Forêt, Université du Québec en Abitibi-Témiscamingue445 boul. de l'Université, Rouyn-Noranda, QC, J9X 5E4, Canada; 3 Département des Sciences Biologiques, Chaire industrielle CRSNG-UQAT-UQAM en Aménagement Forestier Durable, Université du Québec à MontréalP.O Box 8888, Succursale Centre-ville, QC, H3C 3P8, Canada; 4 Ministère des Ressources Naturelles, Direction générale de l'Abitibi-Témiscamingue70 avenue Québec, Rouyn-Noranda, QC, J9X 6R1, Canada

**Keywords:** Abundance, apparent survival, BACI design, Dail–Madsen open *N*-mixture model, *Glaucomys sabrinus*, habitat selection, manipulative experiment, recruitment, snags, tree cavities

## Abstract

Dynamic *N*-mixture models have been recently developed to estimate demographic parameters of unmarked individuals while accounting for imperfect detection. We propose an application of the Dail and Madsen (2011: *Biometrics*, **67**, 577–587) dynamic *N*-mixture model in a manipulative experiment using a before-after control-impact design (BACI). Specifically, we tested the hypothesis of cavity limitation of a cavity specialist species, the northern flying squirrel, using nest box supplementation on half of 56 trapping sites. Our main purpose was to evaluate the impact of an increase in cavity availability on flying squirrel population dynamics in deciduous stands in northwestern Québec with the dynamic *N*-mixture model. We compared abundance estimates from this recent approach with those from classic capture–mark–recapture models and generalized linear models. We compared apparent survival estimates with those from Cormack–Jolly–Seber (CJS) models. Average recruitment rate was 6 individuals per site after 4 years. Nevertheless, we found no effect of cavity supplementation on apparent survival and recruitment rates of flying squirrels. Contrary to our expectations, initial abundance was not affected by conifer basal area (food availability) and was negatively affected by snag basal area (cavity availability). Northern flying squirrel population dynamics are not influenced by cavity availability at our deciduous sites. Consequently, we suggest that this species should not be considered an indicator of old forest attributes in our study area, especially in view of apparent wide population fluctuations across years. Abundance estimates from *N*-mixture models were similar to those from capture–mark–recapture models, although the latter had greater precision. Generalized linear mixed models produced lower abundance estimates, but revealed the same relationship between abundance and snag basal area. Apparent survival estimates from *N*-mixture models were higher and less precise than those from CJS models. However, *N*-mixture models can be particularly useful to evaluate management effects on animal populations, especially for species that are difficult to detect in situations where individuals cannot be uniquely identified. They also allow investigating the effects of covariates at the site level, when low recapture rates would require restricting classic CMR analyses to a subset of sites with the most captures.

## Introduction

Understanding how environmental variables affect spatial or temporal variation in species abundance is one of the main goals of ecological research. Indeed, accurately estimating presence or abundance of a species is usually the most important information required to evaluate the conservation status of a site or to assess the efficacy of management actions (Heink and Kowarik 2010). Analyzing count data without accounting for detection probability can lead to biased abundance and trend estimates (Royle and Nichols 2003; Kéry et al. 2005). To reduce the risk of bias, many monitoring programs now go beyond the use of observed counts as a proxy for true population size (Royle et al. 2004, 2005). Recently developed analytical approaches now enable the estimation of demographic parameters from unmarked individuals (Royle 2004; Dail and Madsen 2011). Such models use count data collected at a number of visits in a given season from a suite of sites, in order to follow temporal variations in population size. These methods show promise in ecology, wildlife management and conservation biology, especially when a limited number of individuals are captured at several sampling sites.

In this study, we examine the value of dynamic *N*-mixture models for understanding the population dynamics of the northern flying squirrel (*Glaucomys sabrinus*), which is of particular interest in North American forest management. The species has been considered an ecological indicator of mature and uncut forests, as well as of boreal forest ecosystem health (Smith 2007, 2012; Holloway and Smith 2011). According to recent studies, occupancy and abundance of northern flying squirrel populations are mostly explained by two key attributes of landscape composition: food and cavity availability. First, food resources may constitute a limiting factor for populations of *G. sabrinus* throughout its range (Ransome and Sullivan 2004; Lehmkuhl et al. 2006; Smith 2007). Conifer trees are known to provide a source of food through seeds and mycorrhizal fungi (Holloway and Malcolm 2006), the most common elements in the diet of *G. sabrinus* (Pyare and Longland 2002). As a result, abundance of this species is often related to the availability of conifer trees (Cotton and Parker 2000; Lehmkuhl et al. 2004; Holloway and Malcolm 2006). Second, tree cavities in the form of dens or nest sites are often found in large-diameter trees or snags of old forests (Holloway and Malcolm 2007a; Smith 2007; Pyare et al. 2010). These cavities constitute the most reliable predictors of microhabitat use and population density of northern flying squirrels in a wide range of habitat types (Holloway and Smith 2011; Smith 2012). However, recent studies using capture–mark–recapture (Lehmkuhl et al. 2006) and occupancy models (Trudeau et al. 2011) accounting for imperfect detectability suggest that highest northern flying squirrel population densities are not always linked to older stands, especially in mixed-wood forests.

Given this lack of consensus in the literature on the importance of mature stands and associated cavities, our main objectives were first, to evaluate the effect of cavity availability on population dynamics of northern flying squirrels through a before-after control-impact (BACI) design consisting of experimental supplementation of cavities between two sampling seasons, and second, to test the application of a dynamic *N*-mixture model in a BACI design. We hypothesized that (1) initial squirrel abundance increases with conifer basal area (indirect measure of food availability – surrogate of seeds and mycorrhizal fungi) and snag basal area (indirect measure of natural cavity availability) and (2) recruitment rate and apparent survival increase with the addition of artificial cavities, particularly where natural tree cavities and food availability are low (interactive effects of nest box addition x snag basal area, and nest box addition x conifer basal area). Finally, to assess the robustness of our results, we compared the estimates obtained from the dynamic *N*-mixture models against single season *N*-mixture models, classic capture–mark–recapture models for closed populations, generalized linear mixed models on unadjusted counts, and Cormack–Jolly–Seber (CJS) models.

## Methods

### Study area and trapping design

We conducted our study in northwestern Québec, in the vicinity of Rouyn-Noranda (48°18′N, 79°05′W) between 2008 and 2012. We selected 56 sites within an area of 100 km^2^, along a gradient of stand age (20–80 years) and cavity availability in even-aged deciduous stands. Trembling aspen (*Populus tremuloides*) was the dominant tree species accompanied by white birch (*Betula papyrifera*), white and black spruce (*Picea glauca*, *P*. *mariana*), balsam fir (*Abies balsamea*), and jack pine (*Pinus banksiana*). All sites were adjacent to an access road, were homogeneous within a 100 m buffer zone, and were separated by at least 400 m from each other to ensure that different squirrels were being sampled and to reduce autocorrelation (home-range around 3 ha, Lehmkuhl et al. 2006; Holloway and Malcolm 2007b).

Each site was first sampled in 2008 and again in 2012 using 8 trapping stations, established along an 80-m linear transect perpendicular to the road (see Trudeau et al. 2011). Stations were separated from each other by 10 m. For each of the two sampling years, we conducted two trapping periods of three consecutive nights between September and December (i.e., for a total of 48 trap-nights per site per year). Traps consisted of single Tomahawk live traps (Model 201; Tomahawk Live Trap Co., Tomahawk, WI), baited with apple wedges and peanut butter. We attached traps to the trunk of trees, alternating between 1.5 m and 4 m above ground level along the 80-m transect. To evaluate the effect of trap height on capture success, we reversed the height attribution at the second trapping period. Metal ear tags were used as a marking method on flying squirrel in 2008 (Model No. 1; National Band and Tag Co., Newport, KY). However, we preferred the use of pit tags in 2012 (HPT9 Biomark, Idaho, USA), mainly to minimize risks of ear injuries. Trapping and all animal manipulation followed the guidelines of the Canadian Council on Animal Care (permits # 2004-03-01 and # 2012-03-07).

### Nest box addition

Flying squirrels can quickly occupy newly available cavities in their environment (Ransome and Sullivan 2004). In December 2010, 29 of 56 sites were supplemented with artificial nest boxes (Junco Technologies Inc.) to increase cavity availability. On each of the 29 sites, we installed 6 artificial nest boxes (19 × 19 × 32 cm) 10 m from the transect, at a height of 4 m. Three nest boxes were placed to the left of stations number 2, 4, and 6, whereas three others were to the right of stations 3, 5, and 7. To simulate heterogeneity in cavity opening, half of the nest boxes had an entrance of 3.81 cm in diameter and the other half, an entrance of 5.08 cm. We visited nest boxes four times after their installation in December 2010, during the daytime each spring and fall thereafter: spring 2011 and 2012 (end of May and beginning of June) and during fall 2011 and 2012 (end of November and beginning of December). On each visit, we inspected the boxes for northern flying squirrels and other cavity users.

### Environmental variables

We characterized habitat variables known to be important predictors of occurrence, density, and nest site selection of *Glaucomys sabrinus*. We considered conifer tree abundance as a measure of potential availability of food (Cotton and Parker 2000; Holloway and Malcolm 2006) and snag abundance as a surrogate of tree cavity availability (Smith 2007; Pyare et al. 2010). Following Patterson and Malcolm (2010), we quantified these attributes at each trapping site based on basal area (m^2^/ha): basal area of living conifer trees (>10 cm diameter at breast height) and basal area of large snags (>20 cm diameter at breast height). Basal area was measured from 3 prism sweeps (basal area factor 2) per site at trap stations number 2, 5, and 8. We also summed the total precipitation for each visit (i.e., 3 days of trapping) as an explanatory variable. Precipitation data were downloaded from the Environment Canada website (http://climate.weathereteo.gc.ca), from the closest meteorological station located in Val-d'Or (48°03′N, 77°47′W).

### Statistical analysis

#### Dynamic *N*-mixture abundance modeling

We included adult and juvenile squirrels in our analysis. Count data were modeled using dynamic *N*-mixture models (Dail and Madsen 2011). Royle (2004) developed single season *N*-mixture models that enabled the estimation of population size at site *i* (*N*_*i*_) and individual detectability (*p*) from unmarked individuals in a population closed to mortality, recruitment, and emigration. He assumed that *n*_*it*_, the number of detected individuals at site *i* on visit *t,* is the result of a binomial process, *n*_*it*_ ∼ Binomial(*N*_*i*_*, p*_*it*_), where *p*_*it*_ is the probability of detecting an individual at site *i* on visit *t*, and the size parameter *N*_*i*_ corresponds to population size at site *i* and follows a Poisson distribution. The dynamic *N*-mixture model is a generalization of the single season *N*-mixture model. It relaxes the closure assumption by describing population change between seasons. Specifically, it includes parameters of initial population state (abundance in first year of sampling (2008), *λ*) and vital rates, namely recruitment rate including births and immigrations (*γ*) and apparent survival (1 – deaths and emigrations, *ω*). The model also describes the observation process underlying data collection (*p*).

The models assumed that (1) there is no change in abundance at the sites between the first and last visit in a given season; (2) covariates can account for detection heterogeneity across time (*t*) and sites (*i*) (e.g., weather variables, habitat variables); (3) detections within each site are independent across visits; and (4) abundance can be modeled by our covariates with an appropriate distribution model (e.g., Poisson, negative binomial, zero-inflated Poisson). Estimates of population size at each time period can be derived from these parameters using a recursive equation of the type *N*_*i,t*_ *= N*_*i,t−1*_ *ω*^*t−1*^ + *γ*(1 − *ω*^*t−1*^)/(1 − *ω*) (Dail and Madsen 2011). In our case, we considered each period of 3 consecutive nights of trapping as a visit in a given season and tabulated the number of unique individuals during each visit. We also assumed that sites were independent, which was plausible as no marked individuals moved between sites during our study. We centered all environmental variables prior to analysis. We did not include variables highly correlated with one another (|*r*| > 0.7) in the same model.

#### Biologic hypotheses

We expected that potential availability of cavity or food would influence initial abundance (*λ*) of flying squirrels in 2008 [*λ*(Snag), or *λ*(Conifer) or *λ*(Snag+Conifer)]. We used the addition of nest boxes as a covariate on recruitment rate (*γ*). We predicted that the effect of adding nest boxes would depend on the natural availability of cavities or food at our sites. Thus, we considered an interactive effect of nest box addition and the availability of cavities or food in the models [*γ*(Boxes), *γ*(Boxes*Snag) or *γ*(Boxes*Conifer)].

Trudeau et al. (2011) reported precipitation and trap height as potential predictors of detection probability. We also suspected a year effect on the probability of detection because of potential cycles in population density (Fryxell et al. 1998). We added Julian day to account for variation in detectability across the season. We developed models with additive and interactive effects of trap height, weather conditions, and years. Finally, we considered habitat effects on squirrel detection. Specifically, we considered the following scenarios on detectability [*p*(Year+ Height+Prec+Jday), *p*(Prec+Jday+Year*Height), *p*(Year*Prec+Year*Jday+height), *p*(Year*Prec+Year*Jday+Year*Height) or *p*(Snag+Conifer)].

In this study, all sites occupied in 2008 were also occupied in 2012 and variables on apparent survival (*ω*) introduced convergence issues. To simplify our models, we considered the probability of apparent survival constant. As the number of parameters in our models was relatively high in comparison with the number of sites, we could not use an all-combinations selection strategy as recommended by Doherty et al. (2012). To avoid over-parameterizing models, we investigated the effect of the variables of interest on a given state or vital rate parameter while holding the others constant (Appendix 1). Our candidate model set included a null model, for a total of 36 models that should influence the abundance of the first season, the recruitment rate, and the detection probability. We ran each model set with the Poisson distribution on abundance and the zero-inflated Poisson distribution because some sites had few squirrel detections, particularly in 2008. We used the unmarked package (Fiske et al. 2012) in R version 2.15.3 (R Core Team 2013) to obtain maximum-likelihood estimates of the parameters. We assessed the goodness of fit of the top-ranked models with the parametric bootstrap using the chi-square as a test statistic with 5000 bootstrap samples. We compared models using the second-order Akaike information criterion (AIC_c_) (Burnham and Anderson 2002; Mazerolle 2013). We used the entire model set to draw our inferences by computing model-averaged parameter estimates (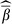) and their unconditional standard errors for the variables appearing in the models with the most support, whereas we model-averaged predictions for the dynamic and detectability parameters from each model (Mazerolle 2013).

#### Comparing dynamic *N*-mixture models with alternative approaches

The top-ranking dynamic *N*-mixture models had marginal fit (see Results). To further investigate the robustness of our results, we tested our hypotheses on each season separately using single season *N*-mixture models (Royle 2004). We tested the effect of potential availability of cavity or food or both on northern flying squirrel abundance in 2008 (i.e., before nest box addition). We also tested the effect of the addition of artificial cavities, alone or in interaction with variables representing cavity or food availability, on northern flying squirrel abundance in 2012 (i.e., after nest box addition). Julian day, trap height, precipitation, and food and cavity availability were tested on detection probabilities for each year. We formulated a total of 12 models for 2008 and 19 for 2012. As above, models were fit with maximum likelihood and compared using AIC_c_ (Burnham and Anderson 2002). We used the same parametric bootstrap approach with 5000 samples to assess model fit.

Despite collecting capture–mark–recapture (CMR) data, we chose the *N*-mixture model approach because sample sizes and recapture rates between periods (4 years) were too low to use with classic CMR models such as Jolly–Seber models for site-specific analyses (Schwarz and Arnason 1996; Williams et al. 2002). For comparative purposes, we pooled the data across all sites and used the Huggins closed population estimator (Huggins 1989, 1991) with two visits to estimate abundance in each year. We considered the effects of conifer basal area, snag basal area, and nest box addition on the probability of capture in different models. Similarly, we built CJS models to estimate annual apparent survival by pooling captures from all sites. These analyses were implemented in a maximum-likelihood framework in program MARK with the RMark package (White and Burnham 1999; Laake 2013). In addition, we also used generalized linear mixed models (GLMM) with a Poisson distribution, log link, and random intercept for each site (Gelman and Hill 2007) to quantify the effects of covariates on counts (estimates of relative abundance) and compare them to the estimates from the *N*-mixture models. Parameters in the GLMM were estimated with the Laplace approximation of the likelihood with the lme4 package (Bates et al. 2013). For the CMR models and the GLMM, we considered a series of candidate models (Appendix 2).

## Results

There were 383 captures (98 for 2008 and 285 for 2012) over 5370 trap-nights. Eighty-three unique squirrels were captured in 2008 and 219 in 2012. We captured squirrels at least once on 33 sites (59%) in 2008 and on 55 sites (98%) in 2012. We captured between 0 and 10 unique squirrels per site in 2008 (mean of 1.5) and between 0 and 8 (mean of 3.9) in 2012. All sites occupied in 2008 were also occupied in 2012 with no extinctions between these 2 years. All sites not occupied in 2008 were colonized in 2012 except one. A single site remained unoccupied in both years (2008 and 2012). The latter was not supplemented with nest boxes and had a total basal area of 20 m^2^/ha and basal area of snags and conifers close to 2 m^2^/ha.

Sites ranged between 7 and 63 m^2^/ha in total basal area (mean = 24 m^2^/ha). Mean basal area of snags was 3.4 m^2^/ha (range 0–13), while mean basal area of conifers was 1.4 m^2^/ha (range 0–6). During autumn trapping, the mean total precipitation for the first visit was 6.2 mm (range 0–16.5 mm) in 2008 and 5.4 mm (range 0.4–18.5 mm) in 2012. For the second visit, the mean total precipitation was 7.3 mm (range 1.0–13.5 mm) in 2008 and 12.2 mm (range 0–42.7 mm) in 2012.

### Nest box visits

The use of nest boxes increased gradually after their installation in December 2010. Only 2% of boxes contained nest material added by squirrels (northern flying squirrels or red squirrels) on the first spring, 26% during the first fall, 36% during the second spring, and 52% during the second fall (after 2 years). Total counts across the entire set of 174 nest boxes (i.e., all boxes combined) ranged between 3 and 27 adult northern flying squirrels in any given year and season. Nine boxes were used by adult female northern flying squirrels with young, whereas only two boxes were used by adult female red squirrels with young. At least one nest box was used in all of the 29 sites where they were added.

### Dynamic *N*-mixture models

Based on the parametric bootstrap, the zero-inflated Poisson distribution provided a considerably better description of the data (*P* = 0.04) than a Poisson distribution (*P* < 0.0002), and the former was used for inference. Two models had most of the support, with a combined Akaike weight of 0.81 (Table 1). These models considered an effect of snag basal area (cavity availability) and conifer basal area (food availability) on initial abundance, no effect of nest box supplementation or surrogates of food or cavity on recruitment rate, and included interactive effects of weather and year on detection probability.

**Table 1:** Top six dynamic *N*-mixture models based on the second-order Akaike information criterion (AIC_c_), showing the distance between each model and the top-ranked model (ΔAIC_c_), Akaike weights (*w*_i_) and number of estimated parameters (K) on the northern flying squirrel data in northwestern Québec during 2008 and 2012

Models	K	AIC_c_	ΔAIC_c_	*w*_i_
*λ*(Snag) *γ*(.) *ε*(.) *p*(Year*Prec+Year*Jday+Height)	12	1038.58	0.00	0.60
*λ*(Snag+Conifer) *γ*(.) *ε*(.) *p*(Year*Prec+Year*Jday+Height)	13	1040.70	2.11	0.21
*λ*(Snag) *γ*(.) *ε*(.) *p*(Year*Prec+Year*Jday+Year*Height)	13	1041.57	2.99	0.13
*λ*(Snag+Conifer) *γ*(.) *ε*(.) *p*(Year*Prec+Year*Jday+Year*Height)	14	1043.83	5.25	0.04
*λ*(.) *γ*(.) *ε*(.) *p*(Year*Prec+Year*Jday+Height)	11	1047.03	8.45	0.01
*λ*(.) *γ*(.) *ε*(.) *p*(Year*Prec+Year*Jday+Year*Height)	12	1049.79	11.21	0.00

### Flying squirrel abundance

**Figure 1:**
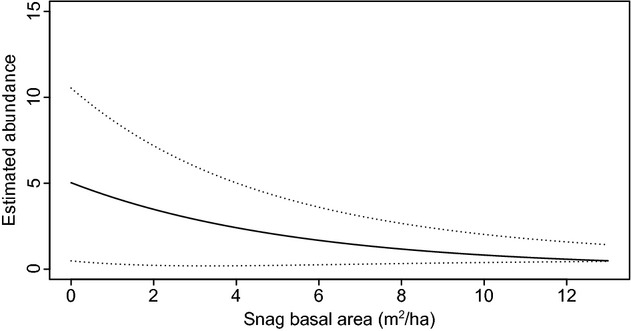
Decreasing abundance of northern flying squirrels in 2008 with the basal area of snags in northwestern Quebec, Canada. Results are based on model-averaged predictions ± 95% confidence limits (dotted lines).

Flying squirrel abundance in 2008 did not increase significantly with an index of food availability (conifer basal area, Table 2). Contrary to our expectations, site-specific abundance in 2008 decreased with the potential availability of cavities (basal area of snags, Table 2), reaching no more than one animal in stands with the highest snag availability (Fig. 1). The model-averaged abundance (± unconditional SE) of flying squirrels for an average site was 2.7 (± 1.47) individuals for 2008 and 7.1 (± 2.1) individuals for 2012 (Table 3).

**Table 2:** Model-averaged parameter estimates for northern flying squirrel abundance in 2008 (*λ*), recruitment rate (*γ*) and detection probability (*p*) in northwestern Québec, Canada, during 2008 and 2012 (apparent survival (*ω*) was considered constant). A 95% unconditional confidence interval excluding 0 indicates that the variable has an effect on a parameter

Parameter	Estimate	SE	Lower 95% CL	Upper 95% CL
Initial Abundance (*λ*)				
Snag basal area	−0.19	0.06	−0.30	−0.08
Conifer basal area	0.09	0.08	−0.07	0.26
Recruitment rate (*γ*)				
Boxes	0.06	0.16	−0.25	0.38
Snag basal area	0.05	0.04	−0.02	0.12
Conifer basal area	−0.11	0.11	−0.32	0.10
Boxes*Snag basal area	−0.04	0.05	−0.14	0.06
Boxes*Conifer basal area	0.18	0.13	−0.07	0.43
Detection probability (*p*)				
Height	−0.28	0.15	−0.57	0.02
Precipitation	−0.07	0.02	−0.12	−0.03
Year	0.54	0.75	−0.94	2.02
Julian Day	−0.02	0.01	−0.03	−0.01
Year*Precipitation	0.07	0.02	0.02	0.12
Year*Height	0.17	0.26	−0.35	0.69
Year*Julian Day	0.02	0.01	0.01	0.04

**Table 3:** Comparison of estimates (± unconditional SE) from dynamic *N*-mixture models, single season *N*-mixture models, generalized linear mixed models, Huggins models, and Cormack–Jolly–Seber models on the northern flying squirrel data in northwestern Québec during 2008 and 2012

		Model type			
		
Parameter	Year	Dynamic *N*-mixture	Single season, *N*-mixture	Huggins	GLMM
Abundance estimate	2008	2.7 (1.47)	2.7 (1.6)	3.2 (0.7)	0.7 (0.1)
	2012	7.1 (2.1)	7.3 (2.5)	5.3 (0.4)	2.4 (0.2)

		Dynamic *N*-mixture			Cormack–Jolly–Seber
Apparent 4-year survival		0.18 (0.25)			0.03 (0.04)

### Flying squirrel recruitment and survival rates

Recruitment rate between 2008 and 2012 did not vary with either the addition of artificial cavities (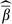 ± unconditional SE: 0.06 ± 0.16), or with indices of natural availability of food (conifer basal area) (−0.11 ± 0.11) or cavities (snag basal area) (0.05 ± 0.04) at our sites (Table 2). Recruitment rate was around 6 individuals per site (95% CI: 3, 12) after 4 years.

Very few northern flying squirrels marked in 2008 were recaptured in 2012 (only 2.4%). Apparent individual survival rate between 2008 and 2012 was considered constant in our models, estimated at 0.18 (unconditional SE: 0.25; see Table 3).

### Detection probability

**Figure 2:**
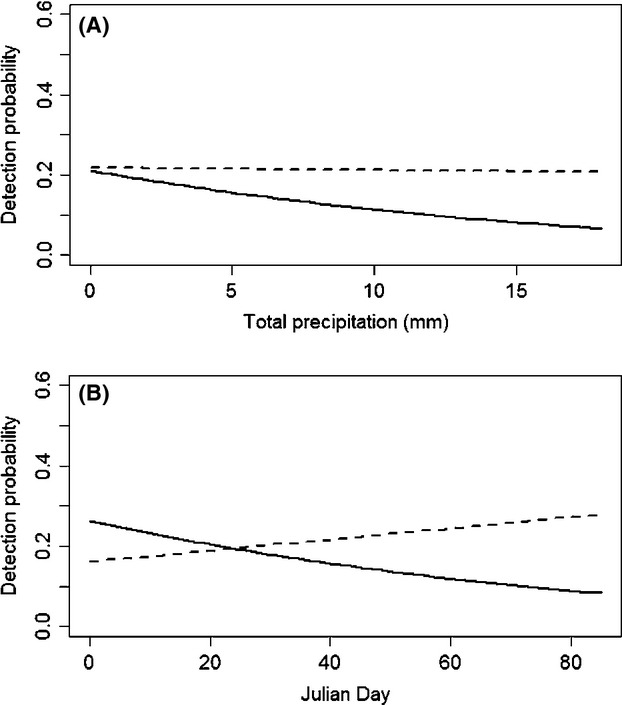
(A) Variation in detection probability of northern flying squirrels in 2008 (solid line) and 2012 (dashed line) with amount of precipitation, in northwestern Quebec, Canada. Results are based on model-averaged predictions. (B) Variation in detection probability of northern flying squirrels in 2008 (solid line) and 2012 (dashed line) depending on Julian Day, in northwestern Quebec, Canada. Results are based on model-averaged predictions.

Detection probability of individuals averaged 0.17, ranging between 0.046 and 0.278 depending on site, precipitation and Julian day. The probability of detection varied with precipitation and Julian day only in some years, with a more negative effect of these variables in 2008 than in 2012 (Fig. 2A,B). Detection probability did not vary with trap height in either year or with habitat characteristics (Table 2).

## Comparison with alternative approaches

### Single season *N*-mixture models

The single season models with a zero-inflated Poisson distribution fit the data well (*P* = 0.57 and *P* = 0.56 in 2008 and 2012, respectively). They led to similar estimated abundance (Table 3) and conclusions to the dynamic *N*-mixture models. Specifically, northern flying squirrel abundance in 2008 decreased from 5 (95% CI: 1, 11) to 1 (95% CI: 0, 1) with the basal area of snags (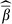 ± unconditional SE: −0.190 ± 0.057).

### GLMM

A single GLMM had the entire support (*w*_i_ = 1). It consisted of the interactive effects of year and snag basal area on counts (Appendix 3). Abundance decreased with increasing snag basal area in 2008 (*β*_Snag_ ± SE: −0.18 ± 0.05). The relationship was weaker in 2012 (*β*_Year x Snag interaction_ ± SE: 0.20 ± 0.05), with a slope of 0.02. There was no evidence of effects of nest box supplementation or conifer basal area on flying squirrel counts as for the dynamic *N*-mixture models.

### CMR models

The closed population models (Huggins) with the most support in both years were those consisting of a time-dependent capture probability (Appendix 3). The capture probability of flying squirrels did not vary with snag and conifer basal areas or with nest box supplementation. Abundance estimates were similar to those of the *N*-mixture models (Table 3).

For the CJS analysis, two models ranked highly compared with the others (Appendix 3). The first model consisted of survival probability constrained to be constant and year-dependent recapture probability, but was followed closely by the model with apparent survival constrained to be equal for intervals of the same length and year-dependent recapture probability. The model-averaged estimate of apparent survival for the period between 2008 and 2012 was 0.03 with an unconditional SE of 0.04 (Table 3).

## Discussion

Four key results emerge from our study. First, initial abundance decreased with an increase in snag basal area (potential availability of cavities), but did not vary with conifer basal area (potential availability of food). Second, recruitment rate and survival probability did not vary with cavity supplementation. Third, the probability of detection varied with precipitation and the advancement in the season (Julian day), but these relationships varied across years. Fourth, *N*-mixture models provided abundance estimates similar to those from classic CMR models, whereas apparent survival from dynamic *N*-mixture models was higher and less precise than reported by CJS models.

### Habitat selection and nest box supplementation

In contrast with our predictions, *Glaucomys sabrinus* apparently does not select deciduous forests with high snag densities. In fact, several sites with high snag densities were not occupied even during the year of low squirrel density. Our results corroborate studies conducted in mixed or in deciduous forests (Wheatley et al. 2005; Patterson and Malcolm 2010). However, our results are also generally in opposition with the importance of snags on squirrel abundance or site occupancy in coniferous forest (Holloway et al. 2012; Shanley et al. 2013).

Our results do not support the claim that the northern flying squirrel is cavity dependent. Flying squirrels use other nest types, such as external leaf nests (dreys) and subterranean structures (Holloway and Malcolm 2007a), especially in fall and winter in deciduous forests (Trudeau et al. 2011). Moreover, this animal is sociable and can share its dens with several conspecifics (Wells-Gosling 1984; Cotton and Parker 2000). Increasing cavity availability does not increase northern flying squirrel population size as shown by nest box supplementation experiments conducted in coniferous (Ransome and Sullivan 2004) or deciduous sites (this study). Based on our own results and on the literature, we conclude that there is no evidence to support that cavity availability is a limiting factor for northern flying squirrels in boreal mixed or boreal deciduous forests.

Studies spanning over several years report annual variation in flying squirrel densities and suggest cycles in population dynamics (Fryxell et al. 1998; Gomez et al. 2005). Lehmkuhl et al. (2006) report density-dependent recruitment for the species. A between-year variation in population levels was also important in our study. Squirrel abundance increased by a factor of 3 in 2012, and individuals were captured at 55 of our 56 sites, regardless of forest composition or nest box supplementation. This population increase may have been related to fungi availability (not directly measured in this study), strong enough to overwhelm the effect of nest box addition. However, even in this case, we would have found more squirrels in most favorable stands (with more food and shelter). Based on these results, *Glaucomys sabrinus* is either opportunistic in terms of its diet, consuming important proportions of insects, plant material, and lichens (Lehmkuhl et al. 2004), or a specialist that moves to find its preferred food when occupying low-quality sites (Lehmkuhl et al. 2006). To further investigate the potential variations of the flying squirrel diet and habitat quality, a project has been initiated to identify food items from DNA extracted from the feces of captured individuals.

The number of individuals captured per 100 trap-nights was exceptionally high for our sites as compared to other studies, especially in 2012 (8.14 in comparison with 3.08 in 2008). Our capture rate was more than four times higher than in other studies: 1.6/100 trap-nights (Wheatley et al. 2005), 2.14/100 trap-nights (Lehmkuhl et al. 2006), or 0.93/100 trap-nights (Patterson and Malcolm 2010). The high recruitment rate from 2008 to 2012 suggests an exponential increase with a good juvenile production within a 4-year period. In 2012, 60% of squirrels captured were juveniles, slightly more than observed by Lehmkuhl et al. in 2006 (52%). The apparent survival probability of individuals for the 4-year interval between sampling seasons was 0.18. This value is consistent with the low number of northern flying squirrels marked in 2008 that were recaptured in 2012. However, as our confidence interval for this estimate is relatively large (0 to 0.8), we must be cautious about these results.

### Detection probability

The detection probability of individuals was relatively low in our case (*P* = 0.17*)*, but very similar to other studies (*P* = 0.18 for Hammond and Anthony 2006, 0.14 for Lehmkuhl et al. 2006). In their occupancy analysis, Trudeau et al. (2011) observed a lower detection probability in high traps than low traps. However, we found no effect of trap height on squirrel individual capture. These discrepancies might stem from the different state variable analyzed (occupancy vs. abundance). Nevertheless, we suggest that the manipulation of trap height, costly in time and logistics in case of high traps, can be abandoned in future studies as they did not translate into a greater capture probability in both types of analyses. Moreover, detection probabilities did not vary among habitat cover types. The lack of consensus in the literature on flying squirrel habitat preference does not seem to be linked to differential detectability across habitat types.

In contrast, precipitation and Julian day negatively affected detection probabilities in 2008 by reducing flying squirrel activity (Trudeau et al. 2011). However, both precipitation and Julian day effects varied with the year, the effects being weaker during the season with higher squirrel density. It is difficult to standardize a trapping study for weather effects when trapping at several sites, along with numerous other factors potentially influencing detectability. In our study area, constant detectability across sites and visits was an unrealistic assumption as often observed for mammals and other taxa (Nichols and Pollock 1983; Williams et al. 2002; Mazerolle et al. 2007). This highlights the importance of estimating detectability in order to obtain meaningful state variables and vital rates, as it renders possible comparison across sites and studies.

### Application of the dynamic *N*-mixture model

Our approach based on dynamic *N*-mixture models is relatively straightforward to implement and can be incorporated into studies spanning several seasons to estimate demographic parameters. These models are particularly well suited for before-after control-impact design studies and could be used in the case of classical environmental impact assessments or to evaluate the effect of management initiatives on animal and plant populations. Although single season *N*-mixture models run separately for each year fit well, dynamic *N*-mixture models have the advantage of including dynamic parameters to describe changes in abundance across seasons.

### Comparison of dynamic *N*-mixture model with alternative approaches

The mean abundance estimates from the *N*-mixture models were similar to those from the classic CMR models, but considerably larger than those from GLMM. However, the precision of CMR abundance estimates was greater than for estimates from *N*-mixture models. This was expected, as CMR data contain more information than counts of unmarked individuals. Although they provided substantially lower abundance estimates than *N*-mixture models, GLMM revealed similar covariate effects. Mixed models do not estimate detection probability explicitly, as the variation in counts due to imperfect detection is partially described by a suite of temporal or spatial random effects (Gelman and Hill 2007; Royle and Dorazio 2008). Surprisingly, *N*-mixture models and GLMM identified a negative effect of snag basal area in 2008 on abundance (*N*-mixture: −0.19, GLMM: −0.18). Given that detectability is not modeled explicitly in GLMM, we expected a weaker relationship than with the *N*-mixture model. Formal evaluation of this pattern through simulations is warranted.

Apparent survival estimated from *N*-mixture models was higher and less precise than that reported from CJS models (Table 3). Assuming constant survival across years, the annual apparent survival estimated by *N*-mixture models would be 0.65 (i.e., 0.65^4^ = 0.18), as compared to 0.41 (i.e., 0.41^4^ = 0.03) using CJS models. Nevertheless, both estimates are similar to the 0.50 reported by Lehmkuhl et al. (2006) obtained from capture–mark–recapture in western interior forests. These survival probability estimates suggest population turnover within a 4 to 5 year interval, agreeing with Fryxell et al. (1998).

Capture–mark–recapture methods are mostly useful when the number of sites is low and the number of individuals captured at each site is large (e.g., >30). Ultimately, the amount of information in the data increases with the number of recaptures. In our case, conducting a classic CMR analysis for each site was not possible and made it difficult to assess the effects of the site-level covariates without pooling sites. The *N*-mixture model approach allowed us to investigate patterns at all 56 sites, instead of restricting our analyses to a subset of sites with the most captures (or captures > 0). Most importantly, it permitted us to quantify the effect of site-specific variables reflecting cover and food availability on abundance and to test the effect of various weather-specific variables on the observation process even when these variables differed across sites.

### Recommendations for management

The lack of transferability of indicator species to other landscapes, ecosystems, or over time is one of the most recurrent problems with the indicator species approach (Lindenmayer and Likens 2011). Results of our study and variations in patterns of the northern flying squirrel habitat selection across habitat types and over time suggest that interregional extrapolation is unjustified. Conclusions derived from western coniferous forests are not directly transferable to deciduous or mixed forests: Our results show that the northern flying squirrel is not a good indicator of specific attributes of old forests, at least in the northeastern part of its range.

## Data Accessibility

The complete data set and R code used in our analyses are available from the Dryad Digital Repository: http://doi.org/10.5061/dryad.p2r8r (Priol et al. 2014).

## Appendix 1: Candidate dynamic *N*-mixture models (Dail and Madsen 2011) for the northern flying squirrel data in northwestern Québec during 2008 and 2012

**Table:**

Models	Description
1. *λ*(.) *γ*(.) *ε*(.) *p*(Year+Prec+Jday+Height)	Null model with additive effects on p
2. *λ*(.) *γ*(Boxes) *ε*(.) *p*(Year+Prec+Jday+Height)	Effect of boxes on recruitment rate with additive effects on p
3. *λ*(.) *γ*(Boxes*Snag) *ε*(.) *p*(Year+Prec+Jday+Height)	Interactive effects of boxes and cavity availability on recruitment rate with additive effects on p
4. *λ*(.) *γ*(Boxes*Conifer) *ε*(.) *p*(Year+Prec+Jday+Height)	Interactive effects of boxes and food availability on recruitment rate with additive effects on p
5. *λ*(.) *γ*(.) *ε*(.) *p*(Year*Prec+Year*Jday+Year*Height)	Null model with interactive effects of year on p
6. *λ*(.) *γ*(Boxes) *ε*(.) *p*(Year*Prec+Year*Jday+Year*Height)	Effect of boxes on recruitment rate with interactive effects of year on p
7. *λ*() *γ*(Boxes*Snag) *ε*(.)	Interactive effects of boxes and cavity availability
*p*(Year*Prec+Year*Jday+Year*Height)	on recruitment rate with interactive effects of year on p
8. *λ*(.) *γ*(Boxes*Conifer) *ε*(.)*p*(Year*Prec+Year*Jday+Year*Height)	Interactive effects of boxes and food availability on recruitment rate with interactive effects of year on p
9. *λ*(.) *γ*(.) *ε*(.) *p*(Prec+Jday+Year*Height)	Null model with interactive effects of year and height on p
10. *λ*(.) *γ*(Boxes) *ε*(.) *p*(Prec+Jday+Year*Height)	Effect of boxes on recruitment rate with interactive effects of year and height on p
11. *λ*(.) *γ*(Boxes*Snag) *ε*(.) *p*(Prec+Jday+Year*Height)	Interactive effects of boxes and cavity availability on recruitment rate with interactive effects of year and height on p
12. *λ*(.) *γ*(Boxes*Conifer) *ε*(.) *p*(Prec+Jday+Year*Height)	Interactive effects of boxes and food availability on recruitment rate with interactive effects of year and height on p
13. *λ*(.) *γ*(.) *ε*(.) *p*(Year*Prec+Year*Jday+Height)	Null model with interactive effects of year and weather on p
14. *λ*(.) *γ*(Boxes) *ε*(.) *p*(Year*Prec+Year*Jday+Height)	Effect of boxes on recruitment rate with interactive effects of year and weather on p
15. *λ*(.) *γ*(Boxes*Snag) *ε*(.)*p*(Year*Prec+Year*Jday+Height)	Interactive effects of boxes and cavity availability on recruitment rate with interactive effects of year and weather on p
16. *λ*(.) *γ*(Boxes*Conifer) *ε*(.)*p*(Year*Prec+Year*Jday+Height)	Interactive effects of boxes and food availability on recruitment rate with interactive effects of year and weather on p
17. *λ*(.) *γ*(.) *ε*(.) *p*(Snag+Conifer)	Null model with additive effects of habitat characteristics on p
18. *λ*(.) *γ*(Boxes) *ε*(.) *p*(Snag+Conifer)	Effect of boxes on recruitment rate with additive effects of habitat characteristics on p
19. *λ*(.) *γ*(Boxes*Snag) *ε*(.) *p*(Snag+Conifer)	Interactive effects of boxes and cavity availability on recruitment rate with additive effects of habitat characteristics on p
20. *λ*(.) *γ*(Boxes*Conifer) *ε*(.) *p*(Snag+Conifer)	Interactive effects of boxes and food availability on recruitment rate with additive effects of habitat characteristics on p
21. *λ*(Snag) *γ*(.) *ε*(.) *p*(Year+Prec+Jday+Height)	Effect of cavity availability on initial abundance with additive effects on p
22. *λ*(Conifer) *γ*(.) *ε*(.) *p*(Year+Prec+Jday+Height)	Effect of food availability on initial abundance with additive effects on p
23. *λ*(Snag+Conifer) *γ*(.) *ε*(.) *p*(Year+Prec+Jday+Height)	Effect of cavity and food availability on initial abundance with additive effects on p
24. *λ*(Snag) *γ*(.) *ε*(.) *p*(Year*Prec+Year*Jday+Year*Height)	Effect of cavity availability on initial abundance with interactive effects of year on p
25. *λ*(Conifer) *γ*(.) *ε*(.)*p*(Year*Prec+Year*Jday+Year*Height)	Effect of food availability on initial abundance with interactive effects of year on p
26. *λ*(Snag+Conifer) *γ*(.) *ε*(.)*p*(Year*Prec+Year*Jday+Year*Height)	Effect of cavity and food availability on initial abundance with interactive effects of year on p
27. *λ*(Snag) *γ*(.) *ε*(.) *p*(Prec+Jday+Year*Height)	Effect of cavity availability on initial abundance with interactive effect of year and height on p
28. *λ*(Conifer) *γ*(.) *ε*(.) *p*(Prec+Jday+Year*Height)	Effect of food availability on initial abundance with interactive effects of year and height on p
29. *λ*(Snag+Conifer) *γ*(.) *ε*(.) *p*(Prec+Jday+Year*Height)	Effect of cavity and food availability on initial abundance with interactive effects of year and height on p
30. *λ*(Snag) *γ*(.) *ε*(.) *p*(Year*Prec+Year*Jday+height)	Effect of cavity availability on initial abundance with interactive effects of year and weather on p
31. *λ*(Conifer) *γ*(.) *ε*(.) *p*(Year*Prec+Year*Jday+height)	Effect of food availability on initial abundance with interactive effects of year and weather on p
32. *λ*(Snag+Conifer) *γ*(.) *ε*(.)*p*(Year*Prec+Year*Jday+height)	Effect of cavity and food availability on initial abundance with interactive effects of year and weather on p
33. *λ*(Snag) *γ*(.) *ε*(.) *p*(Snag+Conifer)	Effect of cavity availability on initial abundance with additive effects of habitat characteristics on p
34. *λ*(Conifer) *γ*(.) *ε*(.) *p*(Snag+Conifer)	Effect of food availability on initial abundance with additive effects of habitat characteristics on p
35. *λ*(Snag+Conifer) *γ*(.) *ε*(.) *p*(Snag+Conifer)	Effect of cavity and food availability on initial abundance with additive effects of habitat characteristics on p
36. *λ*(.) *γ*(.) *ε*(.) *p*(.)	Null model

## Appendix 2: Candidate Huggins models for closed populations, generalized linear mixed models, and CJS models on the northern flying squirrel capture–mark–recapture data in northwestern Québec during 2008 and 2012. Note that to avoid problems of identifiability in the Huggins model, the probability of recapture (c) was constrained to equate to the probability of capture of the last visit

**Table:**

Model type	Model structure	Description
Huggins models^1^	*p*(.)	constant *p*
	*p*(Occasion)	occasion-dependent *p*
	*p*(Snag)	effect of snag basal area on *p*
	*p*(Conifer)	effect of conifer basal area on *p*
	*p*(Boxes)	effect of nest box supplementation on *p*
	*p*(Boxes+Occasion)	additive effects of time and nest box supplementation on *p*
	*p*(Boxes*Occasion)	interactive effects of time and nest box supplementation on *p*
	*p*(Boxes*Snag)	interactive effects of time and snag basal area on *p*
	*p*(Boxes*Conifer)	interactive effects of time and conifer basal area on *p*
Generalized linear mixed models^2^	Intercept only	constant abundance across all sites
	Year	abundance varies with year
	Year+Snag	abundance varies with additive effects of year and snag basal area
	Year+Conifer	abundance varies with additive effects of year and conifer basal area
	Year+Conifer+Snag	abundance varies with additive effects of year, snag basal area, and conifer basal area
	Year*Snag	abundance varies with interactive effects of year and snag basal area
	Year*Conifer	abundance varies with interactive effects of year and conifer basal area
	Year*Boxes	abundance varies with interactive effects of year and nest box supplementation
Cormack-Jolly-Seber^3^	*φ*(Interval) *p*(Year)	apparent survival constrained to be equal for intervals of same length (2 months vs. 46 months) and probability of recapture varies for each year
	*φ*(.) *p*(Year)	apparent survival constrained to be constant and probability of recapture varies for each year
	*φ*(Interval) *p*(Conifer)	apparent survival constrained to be equal for intervals of same length (2 months vs. 46 months) and probability of recapture varies with conifer basal area
	*φ*(Interval) *p*(Snag)	apparent survival constrained to be equal for intervals of same length (2 months vs. 46 months) and probability of recapture varies with snag basal area
	*φ*(Interval) *p*(.)	apparent survival constrained to be equal for intervals of same length (2 months vs. 46 months) and probability of recapture is constant

1 Models involving nest box supplementation were only considered in 2012 (after nest box supplementation).

2 Models were fit with a Poisson distribution, log link, and random intercept for each capture site and included both years.

3 CJS models for unequal time intervals to estimate apparent survival (i.e., *φ*(interval length)).

## Appendix 3: Top-ranked models based on the second-order Akaike information criterion (AIC_c_) for the northern flying squirrel capture–mark–recapture data in northwestern Québec during 2008 and 2012

**Table:**

Model type	Model structure	K	AIC_c_	ΔAIC_c_	*w*_i_
Huggins models with 2008 data	*p*(Occasion)	2	155.93	0	0.99
	*p*(.)	1	166.77	10.8	0.00
Huggins models with 2012 data	*p*(Occasion)	2	468.22	0	0.73
	*p*(Occasion+Boxes)	3	470.21	1.99	0.27
Generalized linear mixed models with both years of data	Year*Snag	5	703.47	0	1
	Year*Boxes	5	716.89	13.42	0
Cormack–Jolly–Seber	*φ*(.) *p*(Year)	3	246.21	0	0.74
	*φ*(Interval) *p*(Year)	4	248.26	2.05	0.26
